# Distillation of essential oils: An innovative technological approach focused on productivity, quality and sustainability

**DOI:** 10.1371/journal.pone.0299502

**Published:** 2024-02-29

**Authors:** Carlos Alberto Tosta Machado, Katharine Valéria Saraiva Hodel, Herman Augusto Lepikson, Bruna Aparecida Souza Machado

**Affiliations:** 1 SENAI CIMATEC, Postgraduate Program in Industrial Management and Technology, SENAI CIMATEC University Center, Salvador, Bahia, Brazil; 2 SENAI CIMATEC, SENAI Institute of Innovation (ISI) in Health Advanced Systems (CIMATEC ISI SAS), University Center SENAI/CIMATEC, Salvador, Bahia, Brazil; Tamil Nadu Dr J Jayalalithaa Fisheries University, INDIA

## Abstract

Essential oil (EO) distillation units, which are commonly installed on farms, have difficultly accessing knowledge centers. The apparent simplicity of the process hides unwanted losses and deviations that go undetected and, therefore, not corrected. This article proposes improvements to the process that are based on “4.0” technologies in order to detect and correct two important deficiencies, with an impact on the yield, quality and environmental performance. The first deficiency comprises the steam channels that are formed through green mass (channeling), are well known and are considered normal by EO producers. Without detection and correction, this negatively affects the extraction results. The second is the lack of technology that is able to automatically determine the extraction endpoint. Smart sensing, control, self-configuration and the dynamic determination of improved process parameters make up a set of actions undertaken by a smart extraction plant (50-liter capacity). Nineteen experiments using lemongrass (*Cymbopogon citratus*) exhibited remarkable 24% and 10% improvements in the yield and quality, respectively. Energy consumption and a more than 50% reduction in the processing complete the set of results achieved. In addition to manufacturing costs and the utilization of capacity, better sustainability indicators are positive consequences of this technological updating.

## Introduction

Essential oils (EO) are aromatic liquids and volatile substances that are obtained via extraction from various parts of plants, such as their flowers, leaves, roots, barks, fruits and seeds. Chemically, they comprise a range of fractions from the terpenoid family that exist in a homogeneous mixture, from the most volatile to the heaviest [1]. Lemongrass (*Cymbopogon citratus)* is one of the plants most used to obtain essential oil due to its sweet and attractive aroma; it is therefore widely applied in the cosmetics and food industry for the development of perfumes, bath soap, tea, and cooking spices, in addition to its use in traditional medicine as an antimicrobial and antioxidant agent [2]. These properties are mainly related to the composition of lemongrass EO, which comprises a significant number of bioactive compounds, such as citral (with two isomers: geranial and neral), myrcene, isoneral, isogeranal, geraniol, geranyl acetate, citronellal, citronellol, germacrene-D, and elemol, in addition to other bioactive and mineral compounds [3]. Noteworthy in this composition is the presence of citral, which is also considered a quality factor of lemongrass EO [4] due to its important bioactive properties [5].

It is common knowledge that the extraction method employed is one of the most critical factors in the process of obtaining EO. Steam distillation is widely applied in the essential oil industry, accounting for more than 93% of the oil volume produced worldwide [1]. Via the use of steam distillation, the technological scenario of the EO extraction industry confers interesting opportunities with regard to enhancements in the yield, quality, energy efficiency, and operational effectiveness [6]. The dimension of sustainability is equally important, as the concern for environmental performance continues to grow. So-called green extraction, which comprises a set of design and operational principles, aims to mitigate this specific area of concern [7, 8]. Usually installed next to farms in rural areas [9], these extraction units are mostly characterized as small and medium-sized companies (SME) that use extremely simple processes and, therefore, represent an interesting opportunity for technological update [10]. The fact that they are far from developed industrial centers means that they experience difficulties when attempting to access innovative technology, both in terms of suppliers and knowledge [11].

Despite its importance in this context, obtaining EO through steam distillation has significant disadvantages, such as its long duration; this can have an impact on the loss of thermosensitive components and reduce the quality of the oil extracted [12]. When duly applied, technological elements that are allied to quantitative and qualitative improvements are able to enhance performance, thus contributing to a minimized energy and water consumption, as well as a reduction in all other manufacturing resources (labor, capacity, maintenance) and the planted area [13]. Industry 4.0 promotes the concept of smart manufacturing and its use as a sustainable process [14]. The significance of applying this technological framework to EO distillation is clear, as it offers operational advantages such as improved yields and quality, as well as reduced production costs and increased product value.

Thus, this study proposes technological improvements to the steam extraction process by targeting two identified weaknesses: channeling detection and correction (or attenuation), and the automatic determination of the process duration, using "industry 4.0" tools to obtain lemongrass EO. The control of the condensation water temperature is also part of the proposal. A long chain of positive results arises from these improvements, beyond the important benefits observed regarding the yield and quality. A better use of capacity, the postponement of investments and enhanced financial and environmental performances are factors that are more important than the operational aspects of the factory floor. For perspective, the environmental performance of the EO industry is indeed not limited to the indicators of energy and water consumption during the process. When there is an improvement in yield, less acreage is needed to produce the same amount of essential oil; this means that fewer supplies for the care of soil, less irrigation water and equipment, and a smaller work force are required.

## Materials and methods

**Fig 1:**
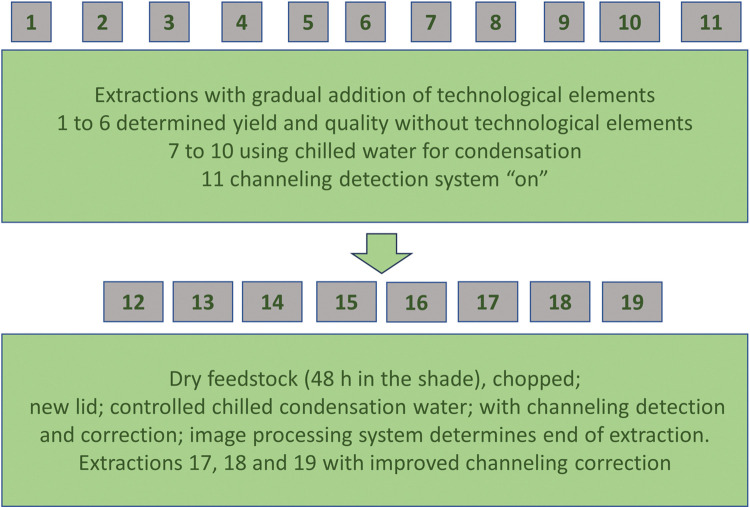
Overview of the 19 experimental conditions employed in this study.

In total, three pillars comprised the scope of this research. The first involved the control of the condensation water temperature, with a focus on an avoidance of losses due to the evaporation of the light compounds. The second involved the detection and control of channeling in order to minimize the risk of temperature overexposure and degradation where the steam channeling takes place, as well increase the yield when steam flows through portions of the raw material that are not fully reached by it. The third involved the use of an image processing system to determine the economical process duration, with several benefits arising from it: shorter extraction times, lower energy requirements, a minimized risk of EO degradation and an improvement in the utilization of capacity. Fig 1 provides an overview of the 19 experimental conditions employed in this study.

### Location

Nineteen experimental extractions of *Cymbopogon citratus* EO were conducted in Morro do Chapéu, a small town in Bahia state, Brazil, in the SME Akã Óleos Essenciais (-11.453386121650805, -41.1775970929219). This company owns a farm in the same location in which the extraction plant is installed (Environmental permit (document 001/2021) and rural property permit (document BA-2921708-BC54.01AE.F2E3.4CF4.B467.B738.6F02.16C5). The environmental permit was granted by the Municipal Department of Environment, Sanitation and Water Resources of the city of Morro do Chapéu, whose purpose is to plan, monitor, supervise and implement policies related to the environment, sanitation and water resources, as well as to implement environmental legislation within the scope of the municipality, with regard to environmental licensing and its supervision. The rural property permit (also called the Rural Environmental Registry) is mandatory in Brazil and is provided by the federal government. One of its purposes is to create a database that facilitates control, monitoring, environmental and economic planning, and the combating of deforestation. No special permits were required to carry out the experimental analyses or to publish the results obtained.

The prompt availability of raw materials is vital for the continuity and success of the experimental. This makes it possible to carry out experiments with plant material supplied from the same origin and with the same agronomic care (fertilization and irrigation). Akã farm is certified as an organic producer (document CA 19303/22). Thus, the use of plant material in this study is in accordance with Akã guidance, as well as national (Brazil) and international guidelines.

### Materials

The plant chosen for the experiments was lemongrass (*Cymbopogon citratus*), which was selected due to its high availability on Akã farm. Such experiments could be performed with any other aromatic plant, as the proposed technologies are intended to be flexible and self-adjusting. The initial extraction was performed with fresh whole leaves (not chopped); this was followed by an extraction with dried whole leaves and another with chopped, dried leaves. The best particle size, according to the authors’ recommendations, was 20 mm [4, 15]. This particle size and the use of dried leaves allowed a higher mass of lemongrass to occupy the internal volume of the extraction vessel (higher mass when weighed fresh).

### Raw material preparation

In order to calculate the yield, the mass of lemongrass was determined immediately after harvesting. For comparison purposes, the initial experimental batches used fresh lemongrass that had not been chopped. Subsequently, trials conducted using dry and chopped plants indicated better yields, confirming the results of previous research. In this work, the yield is expressed in ml/kg, i.e., milliliters of extracted essential oil divided by the mass of fresh raw material (before drying and chopping) fed into the extraction vessel, as shown in Eq 1. For standardization, experimental extractions were performed with lemongrass that was chopped and dried in shade for 48 hours, according to practices established by the research mentioned in the Materials section.


Yield(ml/kg)=Volumeofessentialoil(mL)/Massoffreshrawmaterial(kg) (1)

### Basic extraction equipment

**Fig 2:**
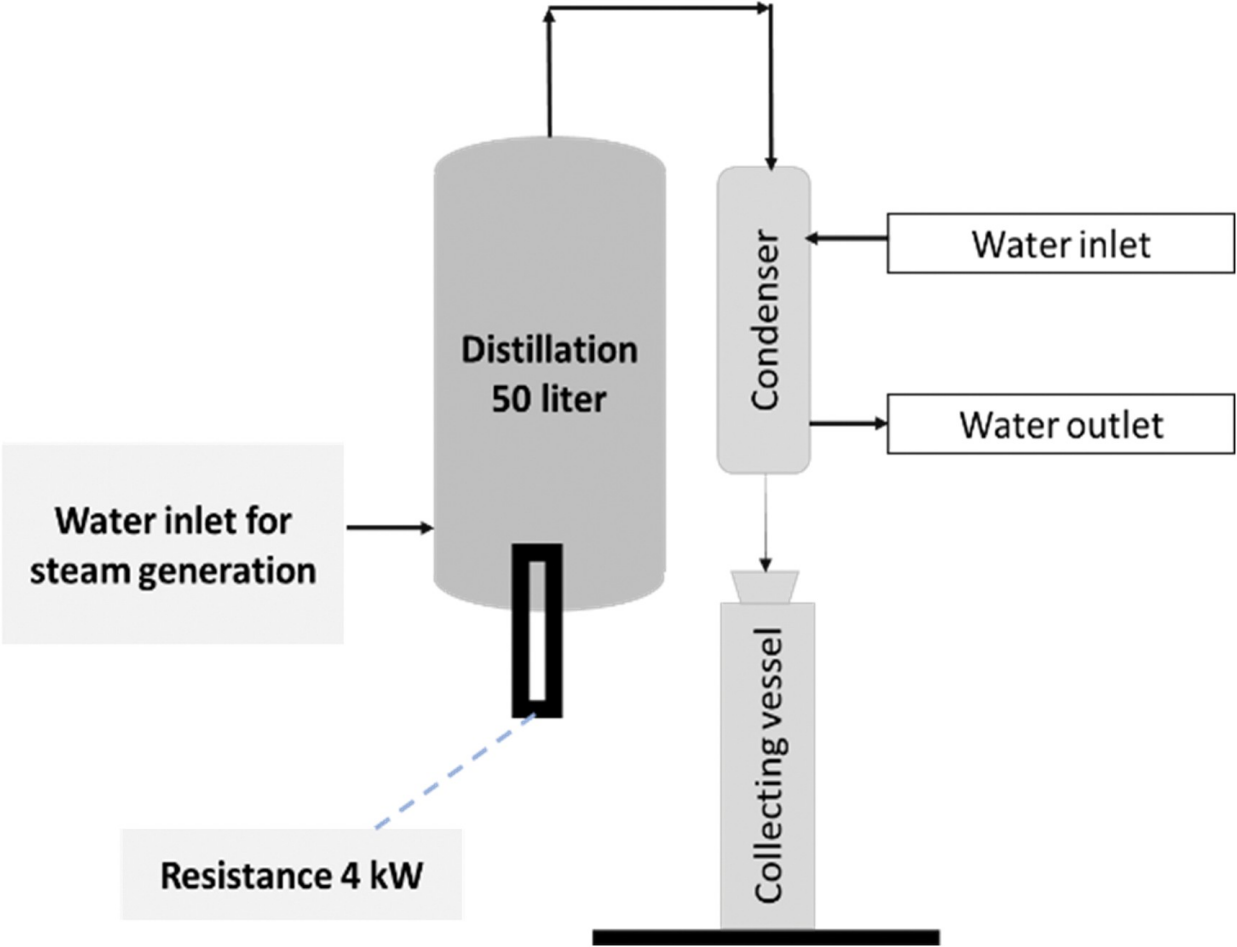
Schematic figure of basic extraction pilot-scale installation. Reprinted from Machado et al. [16] under a CC BY license, with permission from Carlos Machado, original copyright 2023.

The initial experiments were carried out in a pilot-scale plant with a basic arrangement that comprised the following set: (i) electrical resistance: power 4 kW; (ii) distillation vessel: capacity 50L; condenser: tube and shell (iii); and (iv)collecting vessel. Fig 2 represents the mentioned installation. It consists of a plant without sensors and automatic controls, as commonly found in this area of business.

### Basic extraction sequence

Water is introduced into the lower portion of the distillation vessel. The water level is mechanically maintained by a float. After the minimum level has been reached, the resistance (4 kW) starts heating the water to generate steam. A perforated plate that accommodates the raw material is placed immediately above the level of the water. The openings in that plate allow steam to pass through the raw material. After crossing the green matter, the steam carrying the essential oil flows in the direction of the condenser, where liquefaction takes place. The collecting vessel is a transparent tube that receives the mixture and allows the separation of the essential oil and the “water”; this is enabled by the difference in density. That “water” is, in fact, hydrosol, which is a very thin stable suspension that has a low quantity of emulsified essential oil in the condensed water and is difficult to separate.

**Fig 3:**
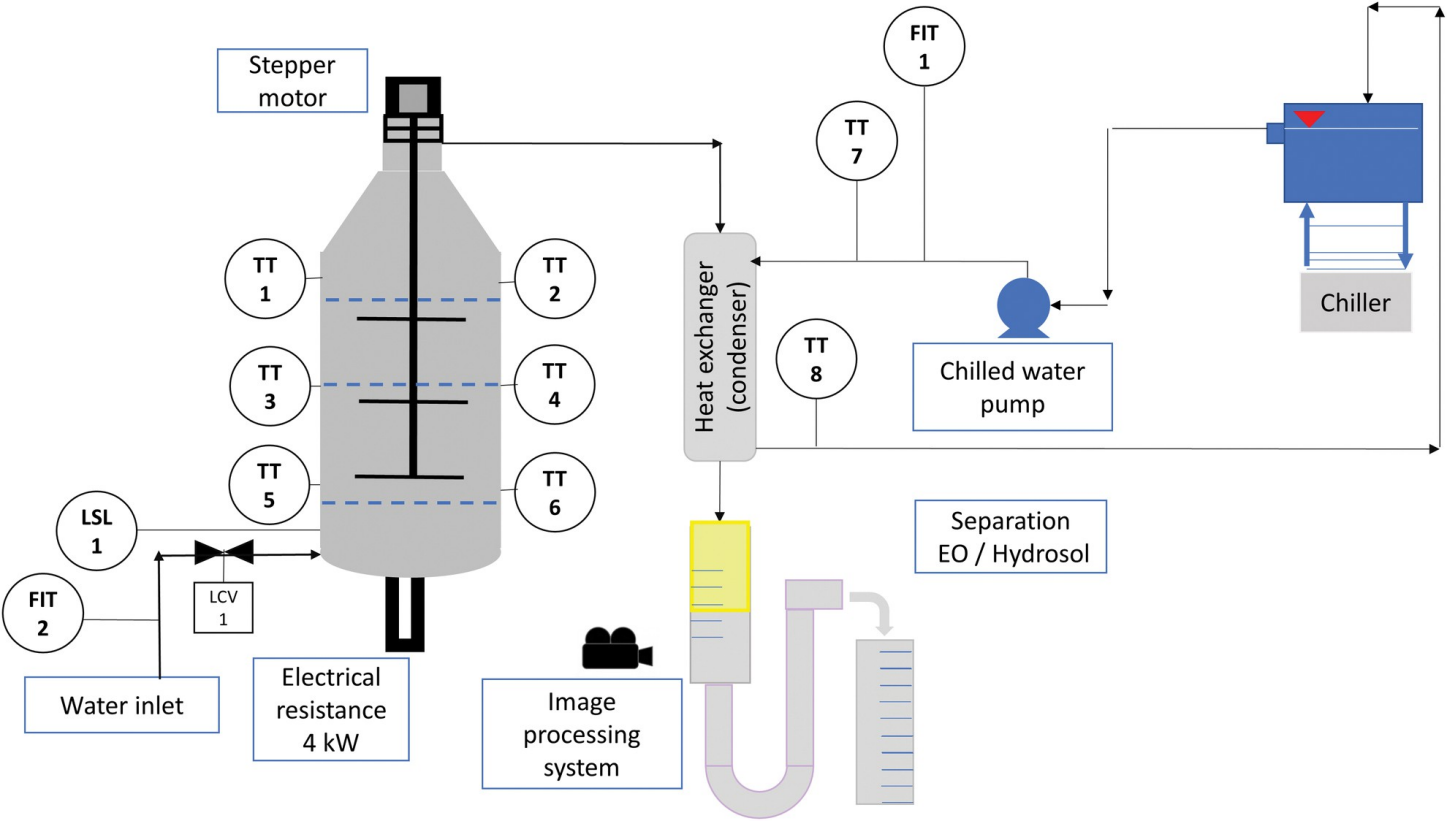
Diagram with instrumentation, chilled water, channeling control, and image processing system. Reprinted from Machado et al. [17] under a CC BY license, with permission from Carlos Machado, original copyright 2023.

The components added over the basic conception, as shown in the Fig 3, are the following:

- TT1 to TT6: Temperature transmitters, thermocouples (type J) inserted directly into the green mass to minimize the time required to transmit the temperature data. The thermocouple wells were not applied.
- Channeling control/Stepper motor: Connected to a thin shaft with slender paddles, this element is designed to promote a re-accommodation of the plant material within the extraction vessel and promote the extinction or attenuation of channeling. The input used to trigger the stepper motor is based on the temperature differences among TT1 to TT6.
- Image processing system: During the extraction, the essential oil column increases until stabilization. In the last period, when the extracted volume is no longer sufficient to pay out the manufacturing costs (energy, water, general operational expenses), the extraction is finished.
- Chiller: The purpose of this device is to supply water with a controlled temperature for condensation, which allows the condensed fluid to be maintained at lower temperatures, thus minimizing the evaporation of the most volatile EO components and avoiding yield losses.

The detection and control of channeling consist of calculating the module of the temperature differences among the six transmitters that are monitored (Eq 2). When this value reaches “T” or higher, an output is sent to turn a preset angle in the stepper motor. This action aims to reaccommodate the raw material inside the extractor vessel, and this routine takes place continuously. However, after an interval (approximately 6 to 8 minutes), the temperatures equalize due to convection and conduction. From this point on, the stepper and motor control logic are changed to a fixed interval (for example, the angle moves 5° every minute or every 45 seconds). It is worth mentioning that several authors note that channeling is a negative event that occurs during extractions; however, this is not evidenced by sensors.

|TTn–TTm|≥T (2)

where TTs are the signal of the thermocouples; T is experimentally determined; and n ≠ m.

**Fig 4:**
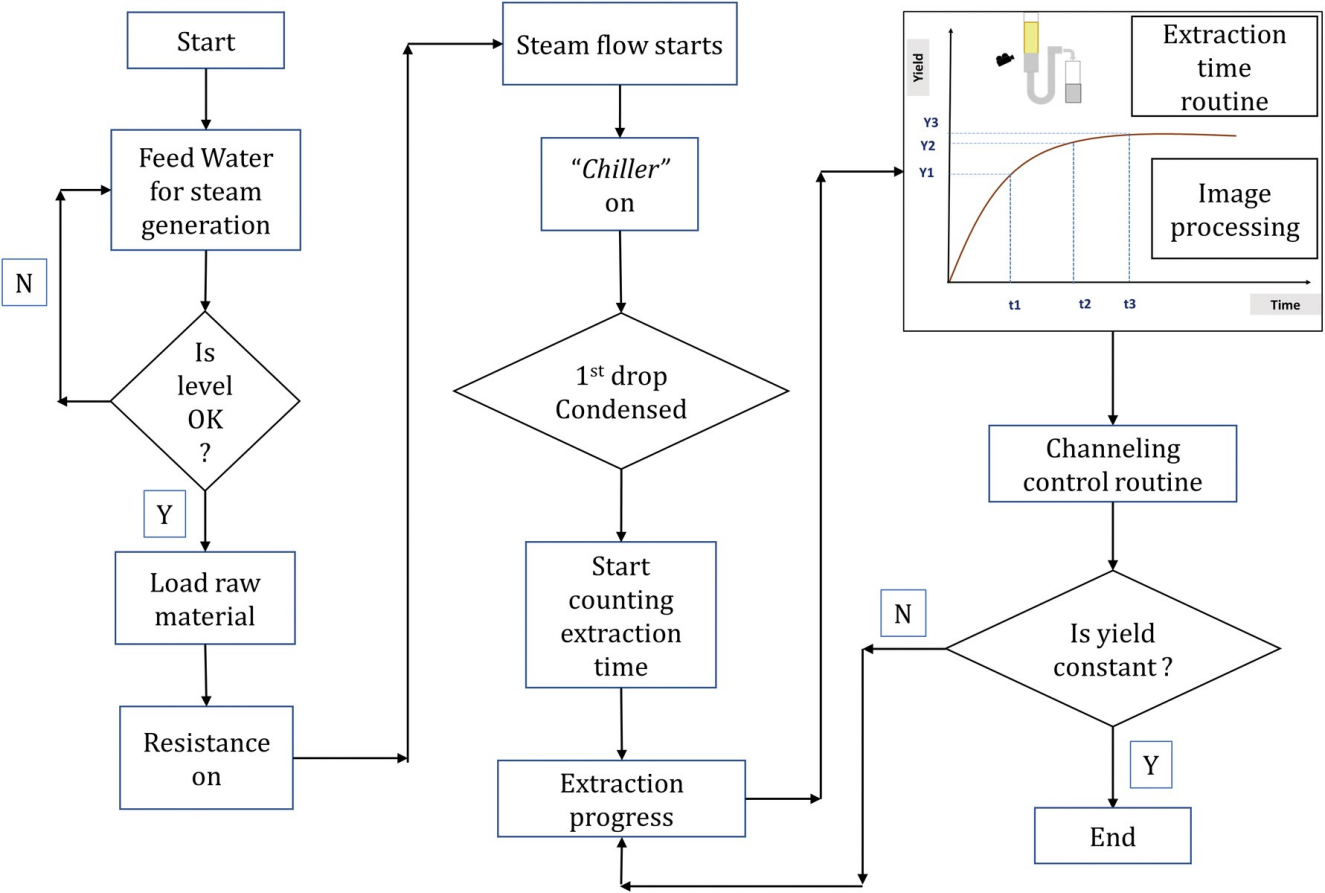
Process flowchart with embedded technology.

Fig 4 outlines a flowchart of the extraction process using the proposed embedded technologies. The routines of the image processing system and channeling control are also shown. Both are run simultaneously until the process endpoint using fixed short intervals. When the first drop of oil–hydrosol is condensed, the measuring of the extraction time is initiated. The extraction is concluded by the image processing system when the maximum extracted volume is reached.

**Fig 5:**
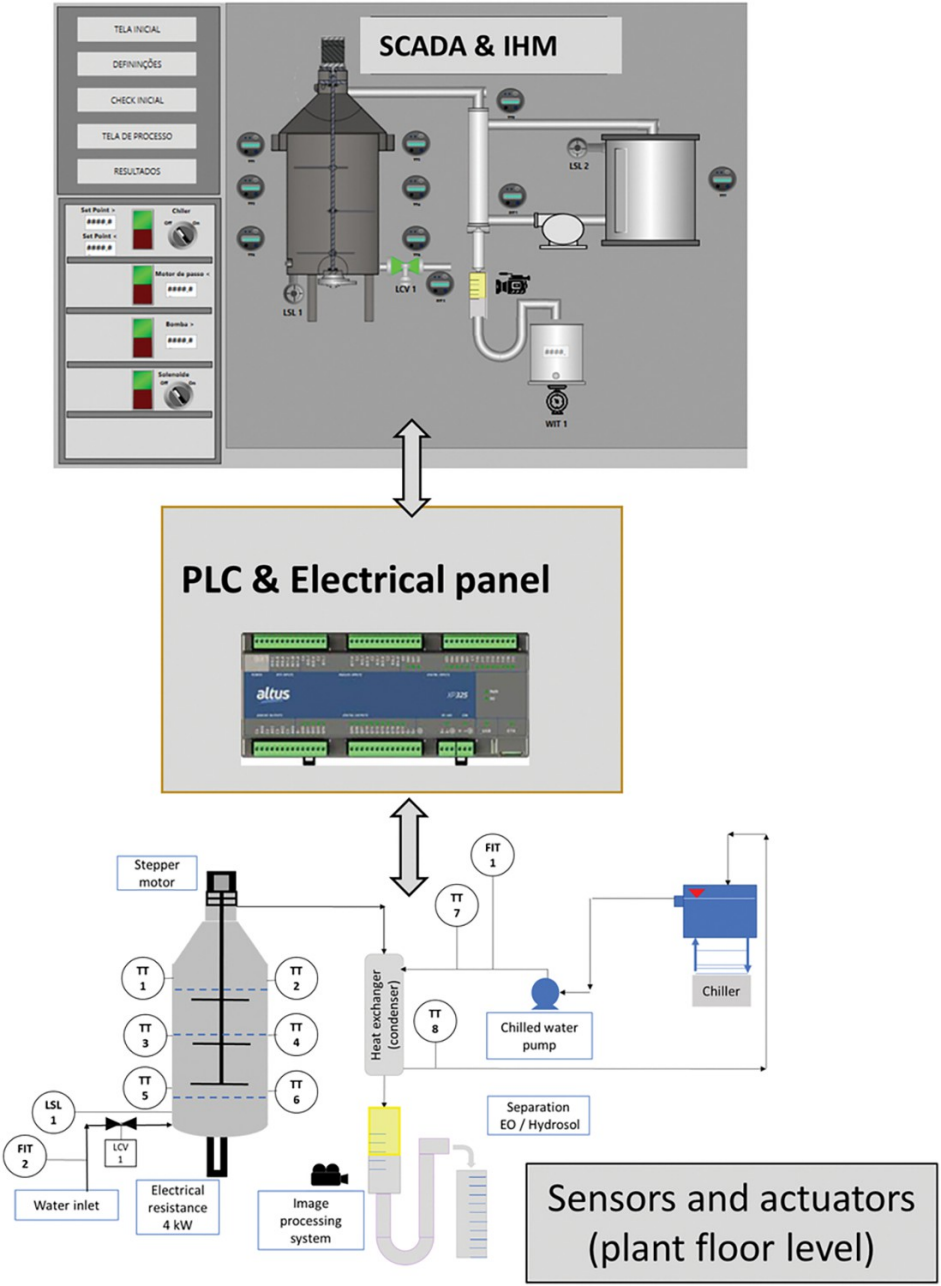
Control architecture: Sensors, image processing system and programmable logic controller (PLC) at plant floor level and remotely installed SCADA (plant floor or control room). Reprinted from Machado et al. [18] under a CC BY license, with permission from Carlos Machado, original copyright 2023.

Fig 5 shows the control architecture responsible for the direct control actions, the data gathering and the man–computer interface. The data analysis enables the CBAs (current best approaches) to be determined for the process parameters. The first control level is a pair of PLCs (programmable logic controllers). The next level is the monitoring system, which is called SCADA (supervisory, control and data acquisition); the process parameters can be input and monitored in this system.

For the image processing system, a camera is connected to a Raspberry controller. Apart from this, all other sensors and actuators employ two Altus PLCs, Nexto Xpress XP340 and XP315 models. A SCADA software called Blueplant, also developed by Altus, 2018 version, was used to create a supervisory application for the essential oil extraction plant.

### Analysis of the bioactive profile of essential oils

Gas chromatography analysis was used to detect the lemongrass (*Cymbopogon citratus*) compounds, using AGILENT 7820A equipment with a flame ionization detector (FID) and injector (split mode at 1:50) at 200°C. The column used was an Alltech EC™wax-10 (30m x 0.32mm x 0.25 μm). The temperature gradient was 50°C (5 min), 3°C/min until 200°C. The detector temperature was 220°C. The volume of EO samples injected was 1 μL at 2.0% m/v, diluted in ethyl acetate. The retention index (RI) utilized was the LTPRI (linear temperature programmed retention index). The validation parameters of the analytical method (such as precision, accuracy, and selectivity) were established based on current Brazilian legislation, especially that linked to the Brazilian Health Regulatory Agency (Anvisa) [19].

### Image processing system

A Raspberry Pi 3 Model B+ controller and its Module V2 camera, coupled to a tripod, were used to capture and process the image; the algorithm was developed in Python language, using the OpenCV computer vision library. This algorithm detects the oil column color via the camera. The user can set the correct color simply by clicking the image. The algorithm can be adjusted to capture the correct color. The data acquired by the PLCs and Raspberry were stored as a process historian within the SCADA. At regular intervals, the SCADA interchanges inputs and outputs with PLCs and Raspberry via the Modbus TCP protocol.

### Data analysis

Nineteen distillation experiments were compared qualitatively and quantitatively; these were particularly considered in terms of the average volume of oil extracted (mL), the yield (mL/kg), and the concentration of the main EO component (citral), which was expressed in the % area in the chromatography analysis.

## Results and discussion

The results will now be discussed, starting with the design of the experimental concept; this was the pilot-scale plant established using embedded technology. The values obtained for the yield and quality evidence the effectiveness of the technological proposal. However, the control of the condensation water temperature, the detection and correction of channeling, as well as the determination of the process’s economical endpoint required the development of an engineered infrastructure to allow these experimental activities.

### Image processing system

#### Channeling detection

**Fig 6:**
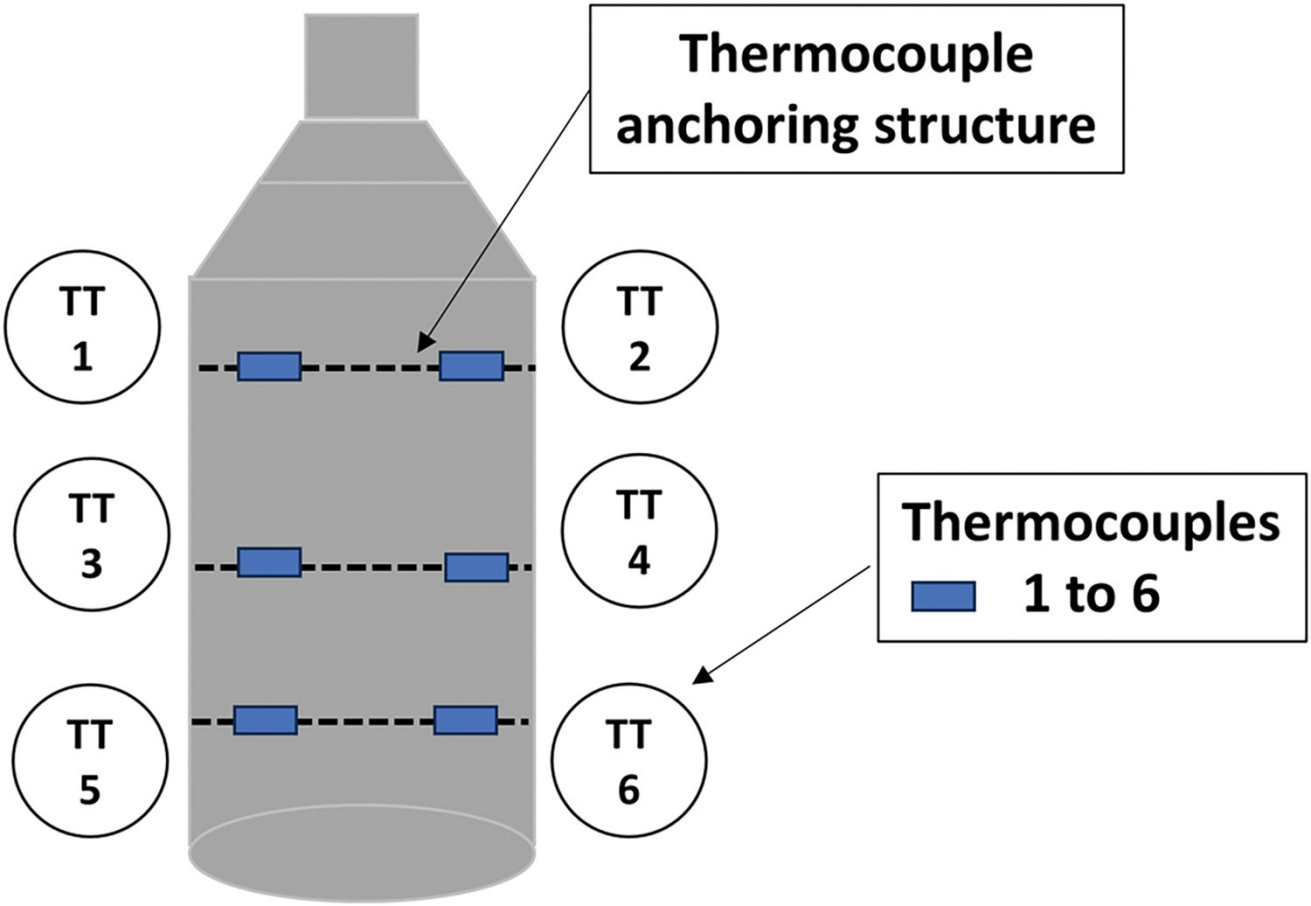
Thermocouple installation: Inserted directly into the raw material mass.

**Fig 7:**
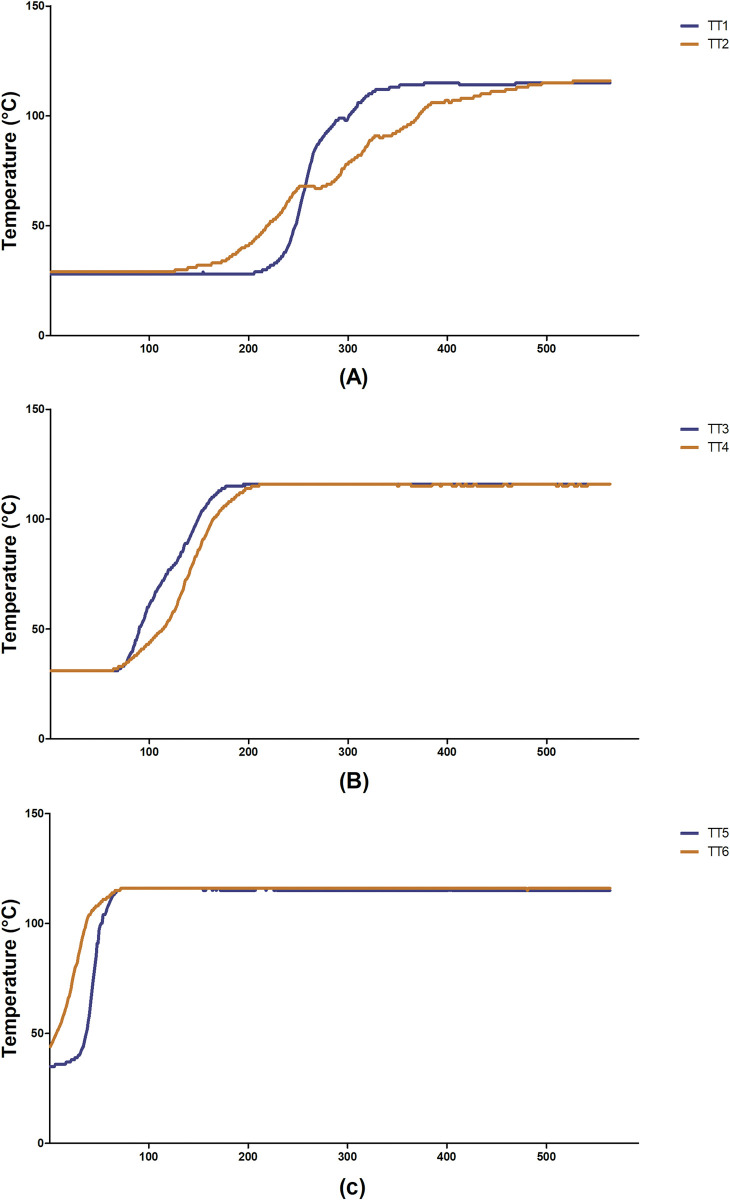
Process temperature (channeling detection) of Experiment 11. (A) TT1 and TT2. (B) TT3 and TT4. (C) TT5 and TT.

Six temperature transmitters, TT1 to TT6, were installed inside the extraction vessel inserted into the vegetable mass in order to detect differences in the temperature. If they occurred, the steam was not flowing evenly through the raw material (green bed), indicating the presence of channeling. The anisotropy of the raw material strongly contributed to the occurrence of channeling during the extraction period. If that difference exceeded a value T, channeling had been detected. In fact, these six thermocouples had to be installed in such a way that they would not interfere significantly with the steam flow. Therefore, they were anchored in a thin wire stainless-steel structure. When two thermocouples installed in the same level indicated different temperatures, channeling had occurred. Fig 6 shows a sketch of the six thermocouples within the extractor. It must be noted that the thermocouple sensors were not installed with the usual thermos-wells. They were placed directly in the raw material in order to reduce the time required to transmit the temperature data. Indeed, the temperature vs. time graphic in Fig 7 shows these differences.

The temperature differences captured by the temperature sensors during the heating period (temperature increase) show that the steam is flowing unevenly through the green mass. This phenomenon is duly registered during the temperature increase; however, it persists even after the temperatures reach the upper level and equalize once the green mass represents an anisotropic medium. Convection and conduction lead to the temperature equalization giving the false impression that channeling was eliminated. The continuous action of the channeling correction system, even after the improvement in the yield and quality as a result of the temperature increase (as shown in extraction results), proves the continuity of the steam streams (channeling). The differences between TT1 and TT2, TT3 and TT4, TT5 and TT6, in a given moment, show that the steam is flowing unevenly; it thus non-uniformly reaches the thermocouples that are installed at the same level of the extracting vessel, which clearly indicates the presence of channeling. In this example (experimental extraction number 11), the steam flow through TT1 and TT2 changes trajectory. It is notable that, after a period, the temperatures are equalized due to convection and conduction.

According to Beis et al. [20], channeling reduces the contact between steam and the raw material, leading to a decrease in yield. Channeling is a phenomenon considered by several authors to be an undesirable occurrence [21, 22]. Nevertheless, its presence is not demonstrated clearly, as shown in Fig 7. Although simple, the installation of temperature sensors allowed this occurrence to be detected. Da Silva et al. [23] found that channeling occurred during experiments that were run in a pilot-scale extraction installation; however, he did not evidence this with, for instance, the installation of sensors.

#### Channeling correction

**Fig 8:**
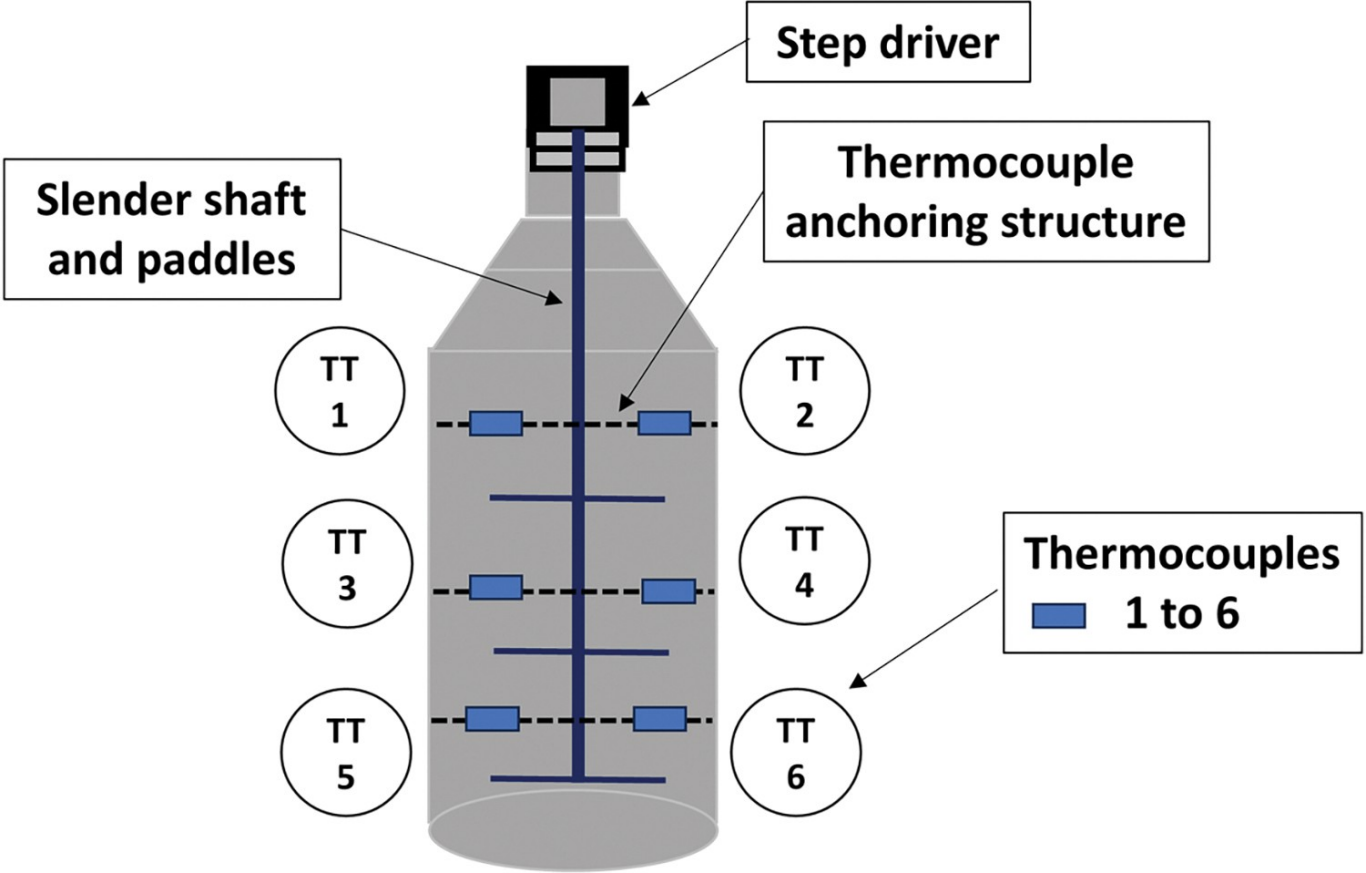
Channeling correction apparat: Thin stainless-steel paddles reaccommodate the herb in order to attenuate or minimize the effects of channeling.

**Fig 9:**
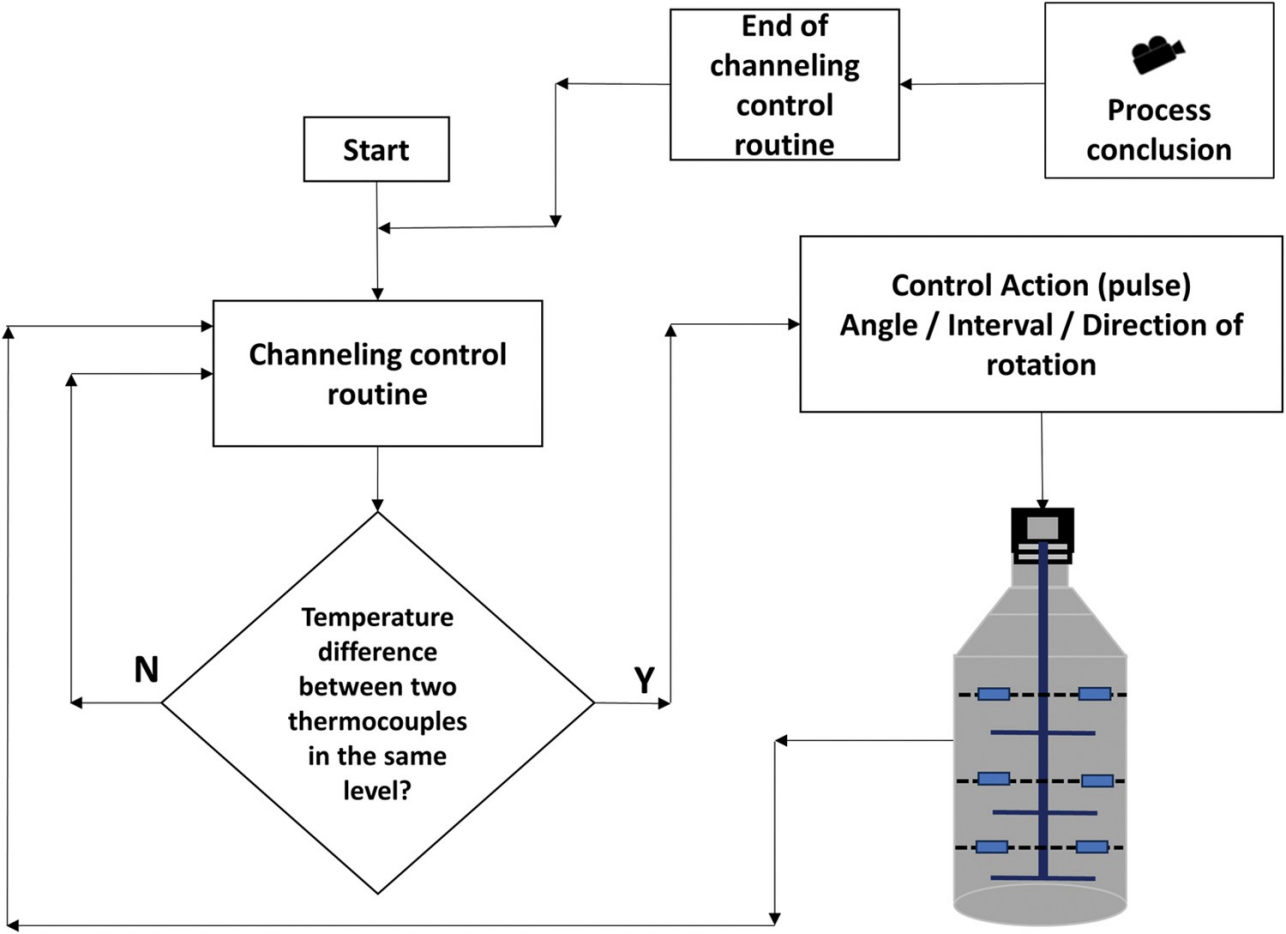
Channeling control flowchart: Temperature differences are the input; control system answers with a step-motor action.

If a difference is detected in the temperature (3 to 5°C), channeling has occurred and a corrective routine takes place. A slim stainless-steel agitator fitted into the extraction vessel, according to Fig 8, performs this correction. Pulses are automatically sent to the step driver of the agitator; this consists of small turning angles that are just enough to reaccommodate the raw material within the extraction vessel, thus breaking or attenuating the channeling. If or when channeling occurs again, the agitator will move, and so forth. These actions are performed to avoid the preferential path of steam through the same portion of green matter and to obtain an even distribution. Nevertheless, after an initial period in which the temperature differences are intense, they equalize due to the heat transfer (conduction and convection). From then on, the channeling control assumes a new routine, with actions occurring every fixed interval (15 to 30 seconds, also subject for the AI learning process). The flow chart shown in Fig 9 illustrates how the detection and correction of channeling were accomplished.

Channeling is a phenomenon that occurs due to the natural anisotropy of green matter (raw material). Despite the operational care taken when feeding and accommodating the feedstock into the distillation vessel, the plant material occupies the internal space in a non-homogeneous manner. Parts of the volume are more compacted than others, even if the raw material is chopped [24]. Researchers have worked with several particle sizes in order to pursue better yields [15, 25]. Yet, steam often finds a preferred route to flow through, resulting in a lower resistance path. As it flows more intensely through these channels, the overexposure of these portions of feedstock to heat result in damage being caused to the product [26]. Nonetheless, whenever the steam flows unevenly through the mass of aromatic plants, the portions not fully reached by it do not contribute to maximizing the yield. Thus, the proper detection and correction of channeling becomes a target for the application of technology with quantitative and qualitative impacts.

The second control logic can be applied at the beginning of the extraction (first condensed drop); however, the detection of channeling via the installation of smart sensors is an important achievement of the present research. Channeling correction (or attenuation) for EO distillation remains a subject of extensive research. The available studies on this matter are not abundant and the results presented in this paper, beyond their importance, may be considered a basis for future progress.

### Determination of economical extraction time

Operationally, the duration of extraction is also of great importance. It becomes a key process parameter when performance, quality, competitiveness and sustainability are at stake. The correct definition of the process times enables higher yields, better quality, the consumption of less energy and water and, very importantly, the appropriate management of capacity [27]. In the essential oil industry, it is common for manufacturers to establish their own extraction procedures and parameters based on informal knowledge regarding the process duration. The extraction time is normally established by the company owner or by an experienced operation leader, and it is kept fixed for future distillations; thus, natural differences in the raw material that are a result of the planting and harvesting conditions are disregarded.

Despite the importance of this factor, there remains a lack of a specific technology to determine the economical extraction endpoint. The research conducted by Benchabane et al. [28] imposed fixed extraction times (30, 60, 120 and 180 minutes), disregarding the productive extraction duration when the essential oil is effectively being extracted. This means that, if the duration is not long enough to complete the extraction, the economical volume is not reached. If longer, the energy and other manufacturing inputs are spent unproductively, not to mention the risk of product degradation due to high-temperature overexposure and the loss of volatile compounds [29].

In this context, the image processing system that is proposed and designed in this work for the detection of the extracted level (and consequently the volume) aims to automatically determine the economical extraction duration. It consists of a camera whose function is to identify the menisci of the oil column, that is, the interfaces between the essential oil and the hydrosol (lower meniscus) and those between the essential oil and the atmosphere (upper meniscus). The essential oil is collected in a transparent tube in which it forms a column that floats on the hydrosol column. Due to the difference in density, there is a separation between the oil, supernatant, and the hydrosol.

**Fig 10:**
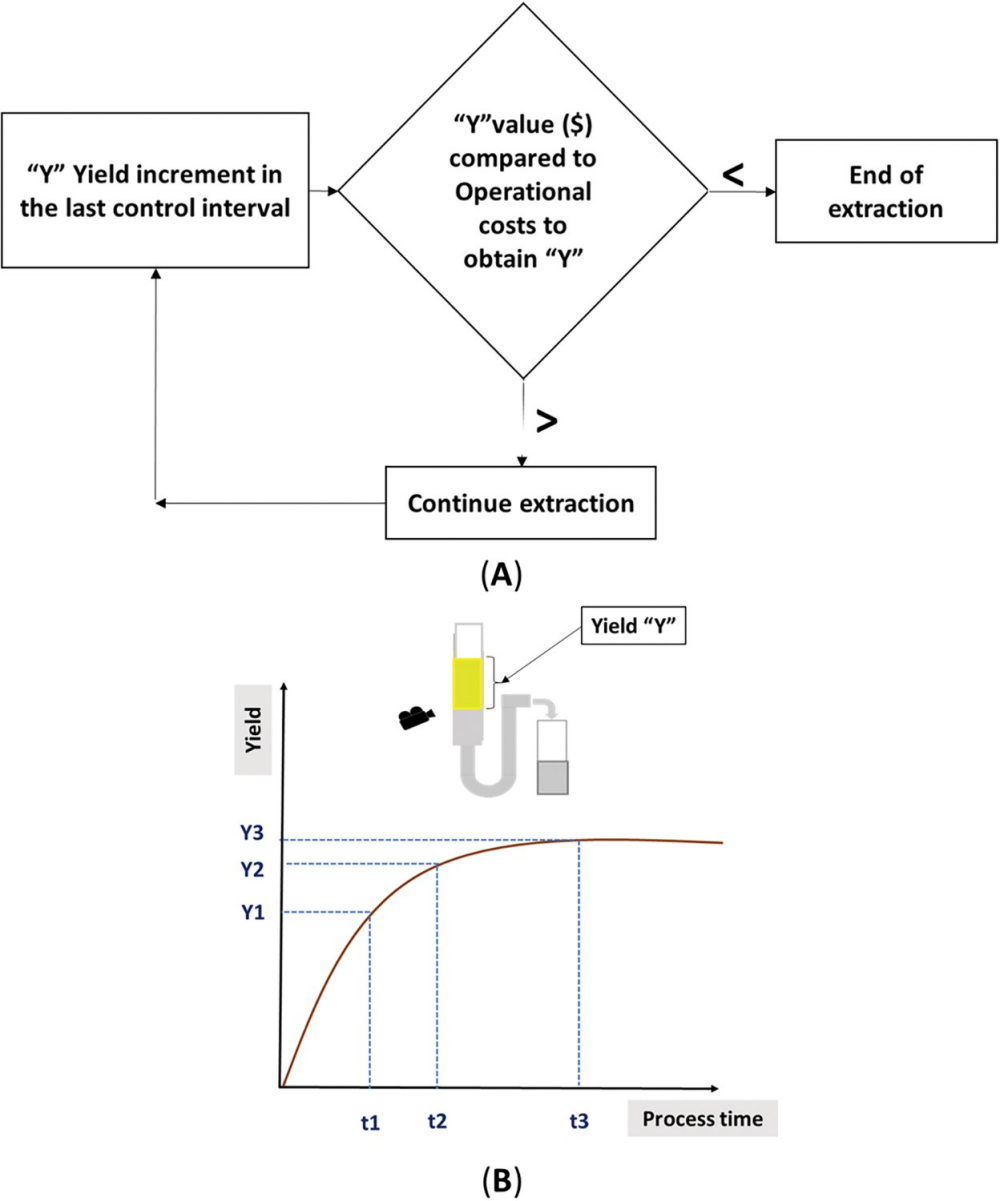
The routine of endpoint determination: After the maximum slope period, the economical endpoint is determined during the asymptotic phase. (A) Routine for the economical extraction time with image processing system. (B) Sketch of the extracted volume versus time graph.

The routine for determining the extraction conclusion point using the image processing system is shown in the flowchart presented in Fig 10A. The yield increment should cover the operational costs involved in producing it; otherwise, the process is not producing real value for the operation and must be stopped. All these routines were performed automatically in this proposed system. Fig 10B is a sketch of a graphic to emphasize the asymptotic behavior of the extraction. In this Figure, T1 represents the end point of the maximum slope in the yield curve. During the interval between T1 and T2, the slope gradually decreases until the curve is flat (practically stable) and no feasible extra volume of EO is extracted. Normally in industry, this process continues until an instant T3 is reached. Note that the EO generated between T2 and T3 would not be sufficient to generate a significant profit margin for the business.

**Fig 11:**
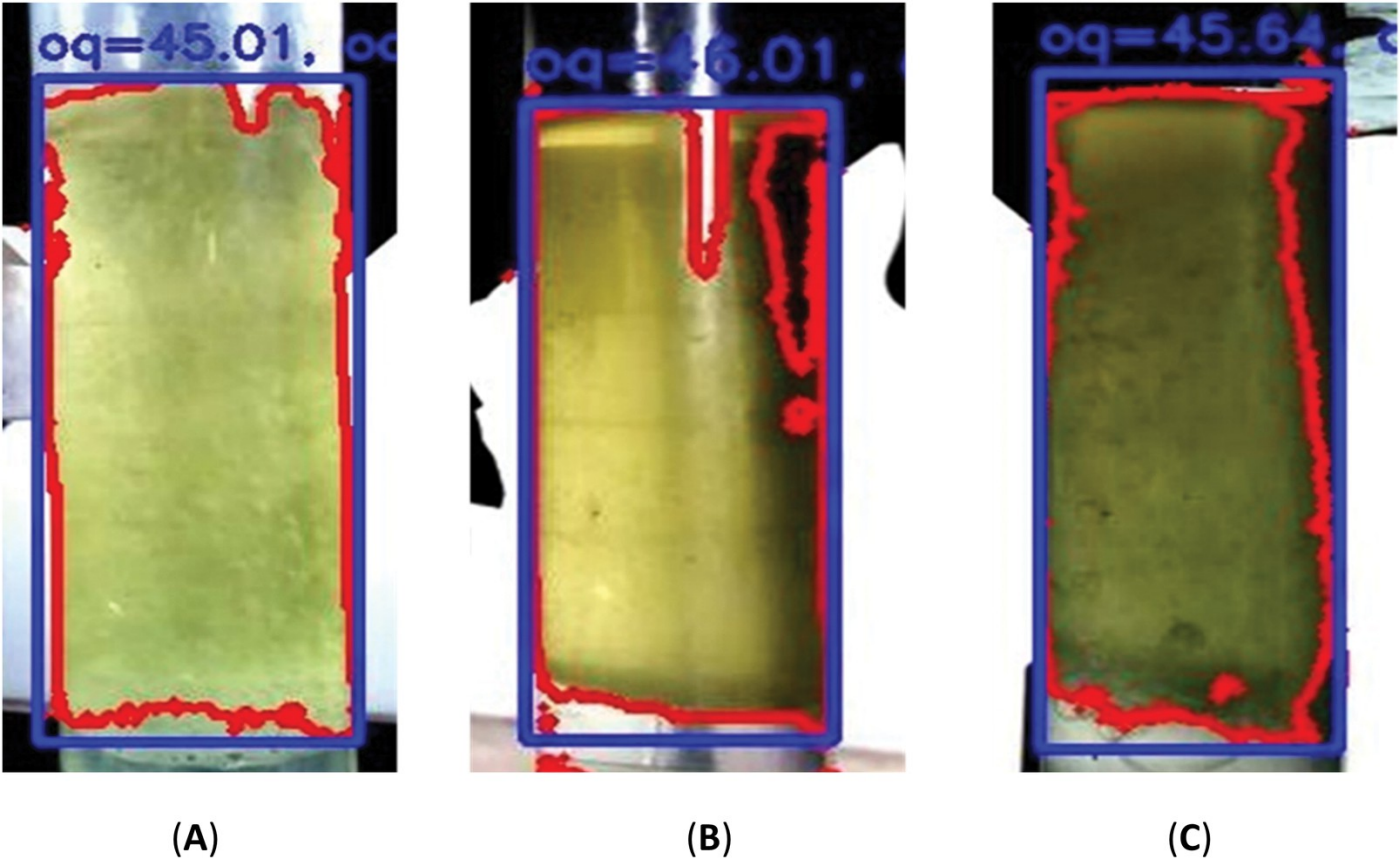
Image of the extraction endpoint. Experiment 17. (B) Experiment 18. (C) Experiment 19. Reprinted from Machado et al. [18] under a CC BY license, with permission from Carlos Machado, original copyright 2023.

Fig 11 shows examples of the images obtained from the extraction endpoint (experiments 17, 18 and 19) when using volumes of 45.01 mL, 46.01 mL and 45.64 mL, respectively. The readings performed (visually) confirmed the value of the image processing system. The advantage of this detection method is its universality; it can be used for any essential oil, even those that are denser than water, by simply reconfiguring the system. A library of colors and patterns can be created so that, when the system is informed of the herb used, it already assumes the appropriate parameters required for detection.

### Yield results

The yield results highlight the importance of adding technology to the steam distillation process [30, 31]. The quantitative improvement was evaluated systematically, according to the implementation of each element; this progress can be noted mainly in the last three experiments, when the average of the extractions reaches 4.60 mL/kg. Table 1 shows the average extraction volume per mode of assembly.

**Table 1:** Average extracted volume per experimental mode.

Experimental mode	Experiments Number	Extracted volume Average (mL)
Manual mode	1, 2, 3, 4, 5, and 6	3.70
Condensation water temperature, chilled, without control, shorter times (with physical observation)	7, 8 and 9	4.08
Condensation water with controlled temperature	10 and 11	4.06
Embedded technology fully implemented	12, 13, 14, 15, 16, 17, 18 and 19	4.33
Embedded technology fully implemented and improved “channeling” routine	17, 18 and 19	4.60

**Fig 12:**
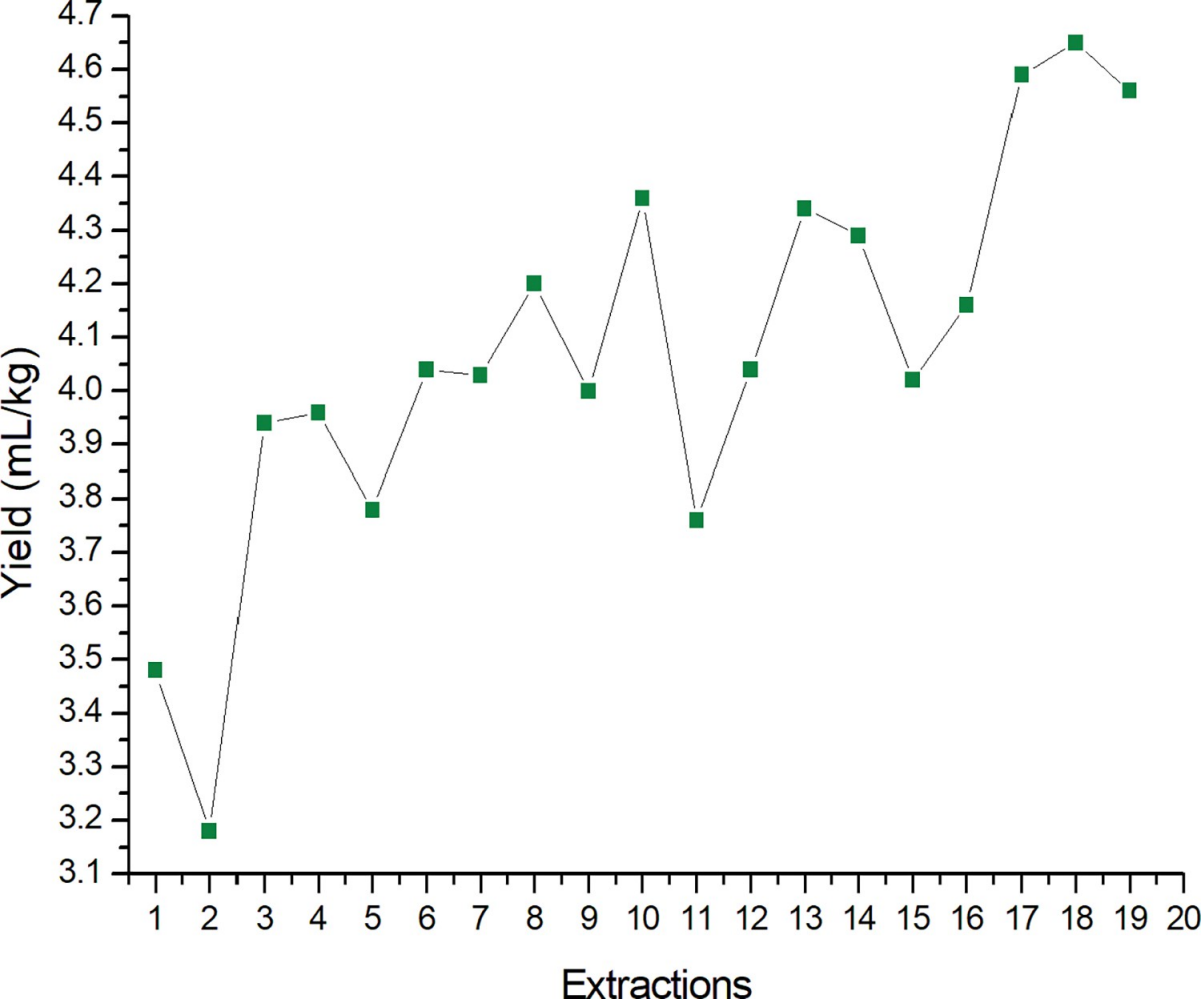
Yield data (mL/kg) of the EO extraction process according to the 19 experimental batches performed.

The results were supported by a series of 19 experiments that were planned in such a way as to enable a comparison between the system without and with imbedded technologic elements to be performed (Fig 12). The yield results highlight the importance of the detection and control of channeling, as well as the positive impact of interrupting the extraction process as soon as the EO column stabilizes. After batch 7, chilled water was used instead of water at room temperature. Rosa et al. [32] evaluated the optimization of the steam distillation process by means of a continuous process; they found that the recovery efficiency rate of an oil obtained from Persian Lime Juice (*Citrus latifolia* Tanaka) was higher than 90%, while the traditional method (discontinuous) showed a recovery efficiency that was between 32 and 60%. Thus, the study conducted by Rosa et al. [32], as well as the results obtained in our work, demonstrate the importance of proposing new methodologies for the optimization of the steam distillation process, especially from an industrial point of view. In addition, because experiments are widely performed at the laboratory scale, it was noted that most of the articles involving the analysis of the EO extraction yield for lemongrass and another vegetal sources are presented as % [33–35]. This mode of presentation was found in the work performed by Boukhatem et al. [36], which reported a yield of 0.6% after 15 minutes of extraction when attempting to obtain EO from lemongrass by means of the solvent-free microwave extraction technique. Nevertheless, the present paper was aimed at the EO industry, where yield is commonly measured in mL/kg.

### Composition of the extracted essential oil: A quality overview

**Fig 13:**
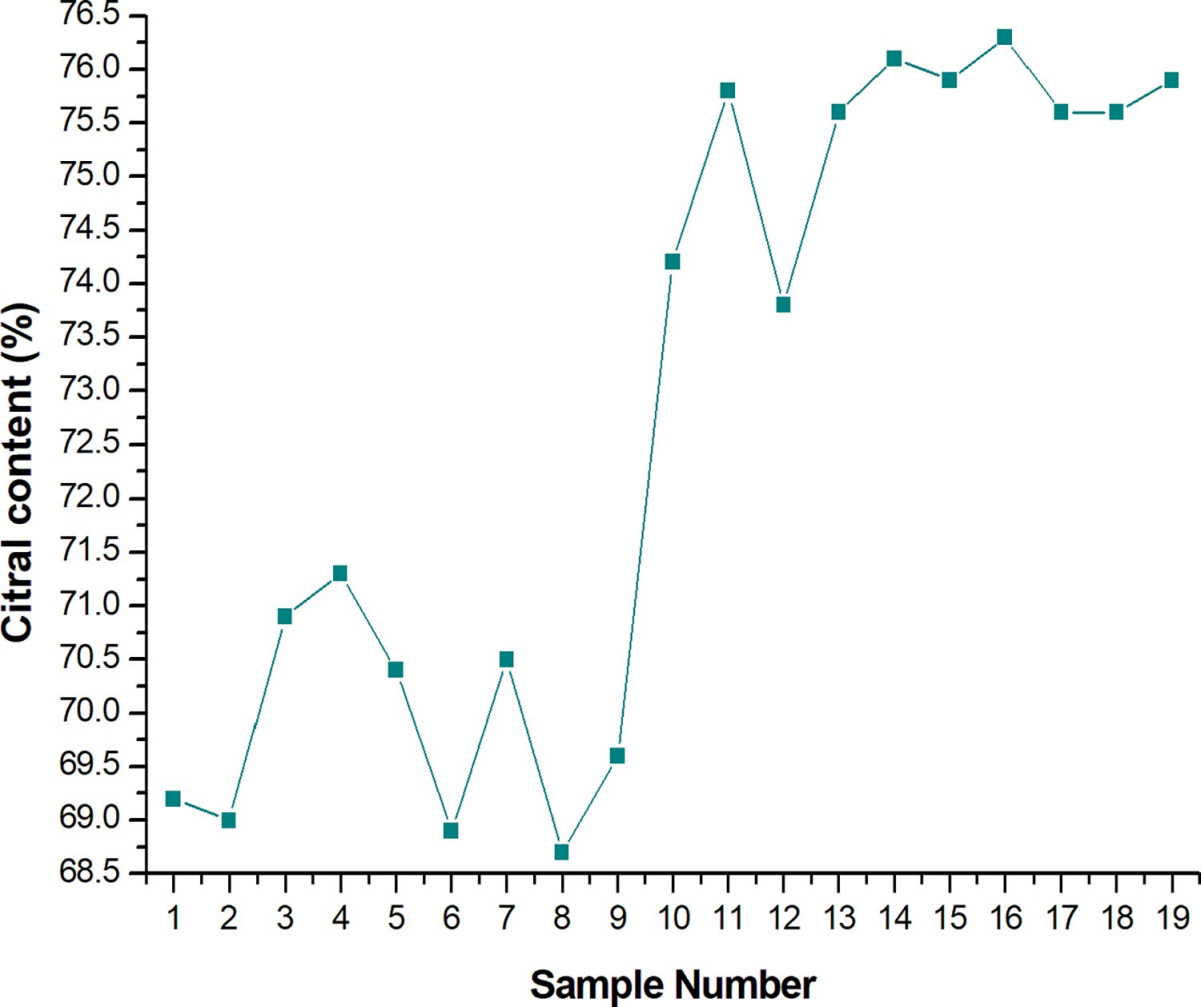
Citral content determined by chromatography for the 19 experimental batches performed.

S1 Table in S1 File shows the chemical composition of the EOs obtained after the implementation of the 19 experimental conditions. Citral is the main component of lemongrass essential oil in terms of its commercial interest, as the value of an essential oil is based on its important components and consequent aromatic properties [37]. S1-S3 Figs in S1 File present chromatograms of experiments 17, 18, and 19, respectively; these experiments exhibited the most promising results among the variables evaluated. After the 19 experimental batches, it may be noted that the citral content increased significantly, from 69% to 76% (Fig 13). The results of the last batches enable the ability of the technology applied to the process to improve the product quality to be affirmed. Specifically, when citral is higher than 60%, it is an indication that (1) the aromatic plant was grown under good plantation practices; (2) the extraction process was efficient; and (3) thermal degradation was minimal, if nonexistent [4]. The citral content in the last experiments show important gains. However, the stability of the citral content in the higher range is an important indicator that the technological elements were indeed effective.

Compared to other studies, it is noteworthy that the results reported by Mahmoud et al. [38] found that the total concentration of citral (geranial + neral) ranged from 57.23% to 66.49% after laboratory-scale extraction using the "Clevenger"-type apparatus. The higher concentration found in the present experiments, when compared to those reported by Mahmoud et al. [38], demonstrate that it is possible to perform extractions in an industrial manner that are of a higher quality than the laboratory-scale experiments. The study by Wan et al. [39] proposed optimizations to the steam distillation process to obtain oil from *Angelicae sinensis* Radix with regard to the concept of quality by design. The oil obtained after the modifications had a Z-ligustilide content, the main component of this oil, that was higher than 85%, which indicates the high quality of the product obtained. The results of Wan et al. [39], as well as those obtained in our study, support the idea that modifications to the process of obtaining essential oils can also result in an improved product quality.

### Environmental performance indicators improvement

The image processing system enables a reduction in the extraction time and thus in the energy and water consumed. Energy and water consumption reductions greater than 50% are directly proportional to the reduction in the process duration. Originally, the process was performed according to the common practices of the industry (2 hours for lemongrass). After the introduction of the image processing technology and the smart determination of the process time, the extraction could reach the maximum volume in 45 to 55 minutes, resulting in a 50% increase in energy and water for steam generation.

Channeling control allied to a shorter extraction time produced a higher yield. When comparing the average of the last three experiments (4.60 mL/kg) with the average yield of the initial trials, not applying the technologies (3.70 mL/kg) resulted in a 24.3% improvement in yield. This advancement reflects upstream with a reduced planted area proportional to the yield gain, demonstrating the environmental success of the present proposal.

The crop yield in Akã, Morro do Chapéu, Brazil, is 1.050 kg in 600 m^2^. These better extraction yields, if extended to the industrial scale, would directly affect this area. For instance, 1.050 kg of lemongrass in the original installation (average yield of 3.70 mL/kg) would provide 3.885 mL, while this weight of lemongrass in the updated installation would deliver 4.830 ml. The planted area required to produce 3.885 mL in the proposed (updated) installation would be 482.6 m^2^, reflecting a reduction in all elements relating to agricultural care and the utilization of irrigation water.

## Conclusions

The technological approach proposed in this work addresses three aspects of the process of extraction using distillation: condensation water temperature control, the correction or attenuation of channeling, and the determination of the economical point of extraction. This technology certainly offers a multitude of other possibilities, such as studying the extension of these improvements to the industrial scale. In fact, the benefits of this progress are not limited to the yield and quality obtained; they may also touch the realms of environmental care and sustainability once these gains are closely related to the upstream material supply chain.

## Supporting information

S1 File (PDF)
