# Supplementary material for: Distillation of essential oils: An innovative technological approach focused on productivity, quality and sustainability
Source: PLoS One. 2024 Feb 29;19(2):e0299502. doi: 10.1371/journal.pone.0299502 (PMC10903824; doi:10.1371/journal.pone.0299502)
Supplement: S1 File — (PDF) [file pone.0299502.s001.pdf]

Manuscript*:* Distillation of essential oils: an innovative technological approach focused on productivity, quality and sustainability

Authors: Carlos Alberto Tosta Machado, Katharine Valéria Saraiva Hodel, Herman A. Lepikson and Bruna Aparecida Souza Machado

Supplementary Material

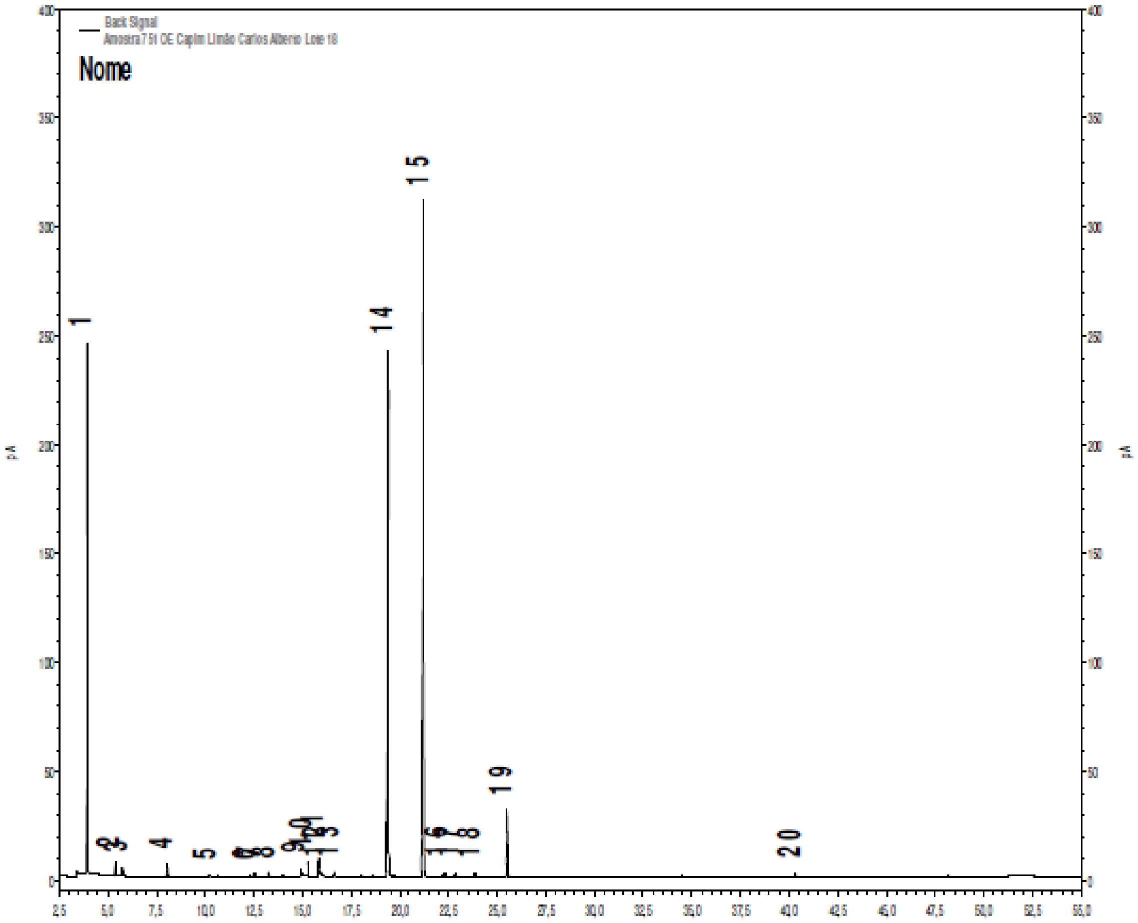

S1 Fig. Chromogram of experiment 17.

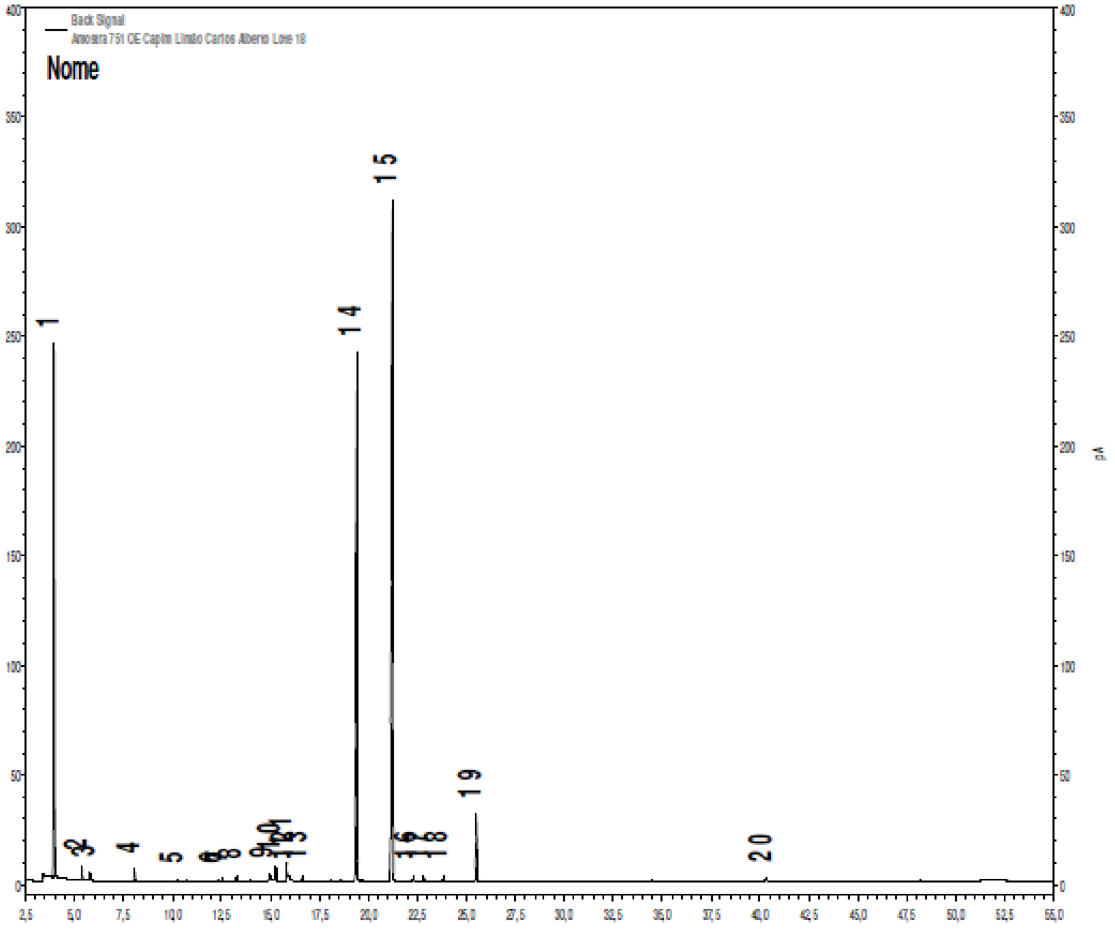

S2 Fig. Chromogram of experiment 18.

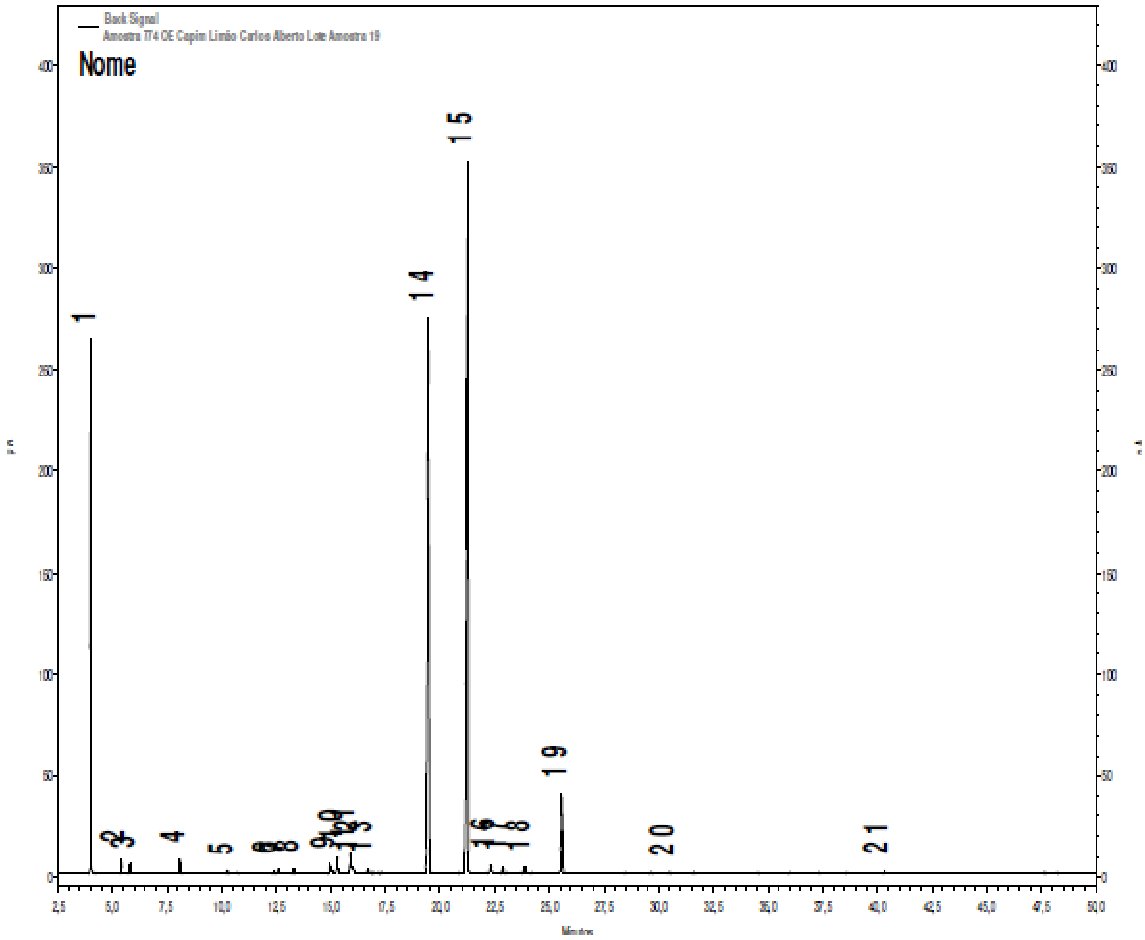

S3 Fig. Chromogram of experiment 19.

S1 Table. Chemical compositions (%) of the lemongrass oil by 19 conditions.

(Area) Percentages present in the analysis (%)																				
Iten	Compound	Experiments																		
		1	2	3	4	5	6	7	8	9	10	11	12	13	14	15	16	17	18	19
1	myrcene	16.0	18.4	15.8	15.6	16.4	15.1	17.1	16.7	17.5	14.7	13.2	15.4	11.6	13.6	12.3	13.3	14.0	14.0	12.8
2	Z-β-ocimene	0.5	0.6	0.5	0.5	0.5	0.5	0.5	0.5	0.6	0.5	0.5	0.5	0.4	0.4	0.4	0.4	0.4	0.4	0.4
3	E-β-ocimene	0.4	0.4	0.4	0.4	0.4	0.3	0.4	0.3	0.4	0.3	0.3	0.4	0.3	0.3	0.3	0.3	0.3	0.3	0.3
4	6-metil-5-hepten-2-ona	0.9	0.7	0.5	0.7	1.0	0.7	1.2	1.2	1.4	0.6	0.5	0.9	0.4	0.4	0.4	0.4	0.5	0.5	0.5
5	6.7-epoxymyrcene	0.1	0.1	0.1	0.0	0.1	0.0	0.1	0.2	0.2	0.2	0.2	0.1	0.2	0.2	0.1	0.2	0.2	0.2	0.2
6	limonene oxide	0.2	0.2	0.1	0.1	0.2	0.0	0.2	2.0	0.2	0.1	0.1	0.2	2.4	0.1	0.1	0.1	0.1	0.1	0.1
7	citronellal	0.3	0.2	0.2	0.2	0.2	0.2	0.2	0.2	0.2	0.2	0.2	0.2	0.2	0.2	0.2	0.2	0.2	0.2	0.2
8	cis-sabinene hydrate	0.3	0.3	0.3	0.3	0.3	0.2	0.3	0.3	0.3	0.3	0.3	0.3	0.3	0.3	0.3	0.2	0.2	0.2	0.2
9	camphor	0.9	0.7	0.8	0.9	0.9	0.9	1.1	1.0	1.1	0.9	1.0	0.8	0.9	1.1	0.8	0.9	1.0	1.0	0.9
10	α-gurjunene	1.0	1.0	0.9	1.0	1.1	0.9	1.1	1.0	1.0	0.8	0.8	0.9	0.8	0.8	0.8	0.7	0.7	0.7	0.7
11	β-elemene	1.0	0.8	0.8	0.9	1.0	0.9	1.0	1.0	1.2	0.9	1.0	0.8	1.0	1.1	0.9	1.0	1.1	1.1	0.9
12	linalool	0.5	0.6	0.7	0.8	0.6	0.8	0.6	0.6	0.7	0.5	0.5	0.5	0.5	0.6	0.6	0.6	0.6	0.6	0.5
13	myrcenol	0.6	0.6	0.6	0.6	0.6	0.6	0.6	0.6	0.5	0.3	0.3	0.4	0.3	0.3	0.3	0.2	0.2	0.2	0.2
**14**	**neral**	**29.4**	**29.6**	**29.8**	**30.5**	**30.2**	**29.7**	**30.6**	**29.5**	**30.5**	**31.5**	**31.9**	**31.8**	**31.9**	**32.0**	**32.0**	**32.3**	**32.0**	**32.0**	**32.1**
**15**	**geranial**	**39.8**	**39.4**	**41.1**	**40.8**	**40.2**	**39.2**	**39.9**	**39.2**	**39.1**	**42.7**	**43.9**	**42.0**	**43.7**	**44.1**	**43.9**	**44.0**	**43.6**	**43.6**	**43.8**
16	α-farnesene	0.6	0.3	0.4	0.4	0.4	0.3	0.3	0.2	0.2	0.2	0.2	0.3	0.3	0.2	0.4	0.4	0.3	0.3	0.5
17	γ-cadinene	0.3	0.3	0.2	0.3	0.3	0.2	0.3	0.3	0.3	0.2	0.3	0.3	0.2	0.3	0.4	0.3	0.3	0.3	0.3
18	nerol	0.7	0.8	0.8	0.9	0.9	0.8	0.6	0.7	0.6	0.4	0.4	0.4	0.4	0.4	0.4	0.4	0.3	0.3	0.3
19	geraniol	4.8	3.9	4.0	3.9	3.7	4.0	3.4	3.0	3.4	3.6	3.6	3.6	3.4	3.1	3.1	3.6	3.7	3.7	4.0
20	cubenol	0.0	0.1	0.0	0.0	0.0	0.0	0.0	0.1	0.0	0.0	0.0	0.0	0.1	0.0	0.0	0.0	0.0	0.0	0.0

21	α-cadinol	0.0	0.1	0.3	0.3	0.3	0.3	0.0	0.1	0.0	0.2	0.1	0.1	0.2	0.2	0.3	0.2	0.2	0.2	0.2
22	caryophyllene oxide	0.0	0.0	0.1	0.0	0.0	0.1	0.0	0.1	0.0	0.1	0.0	0.0	0.0	0.0	0.0	0.0	0.0	0.0	0.1
23	T-muurolol	0.0	0.0	0.1	0.0	0.0	0.1	0.0	0.0	0.0	0.0	0.0	0.0	0.0	0.0	0.0	0.0	0.0	0.0	0.0
24	(E.Z)-farsenol	0.0	0.0	0.1	0.0	0.0	0.3	0.0	0.0	0.0	0.0	0.0	0.0	0.0	0.0	0.0	0.0	0.0	0.0	0.0
25	α-bisabolol	0.0	0.0	0.0	0.0	0.0	0.0	0.0	0.0	0.0	0.0	0.1	0.0	0.1	0.0	0.0	0.0	0.0	0.0	0.0
	other	1.6	1.0	1.1	1.0	0.9	3.8	0.4	1.2	0.5	0.7	0.4	0.2	0.5	0.4	1.7	0.2	0.1	0.1	0.7
